# Interferon-α adjuvant therapy decreases the recurrence of early clear cell renal cell carcinoma and improves the prognosis of Chinese patients

**DOI:** 10.18632/oncotarget.18567

**Published:** 2017-06-19

**Authors:** Hang Yin, Cheng-Gong Liao, Jian-Guo Huang, Yong-Qiang Wang, Zheng Li, Lu-Lu Fan, Men-Long Qian, Nao Wan, Ning Lu

**Affiliations:** ^1^ Department of Oncology, General Hospital of Xinjiang Military Command, Urumqi 830000, P.R. China

**Keywords:** clear cell renal cell carcinoma, interferon-α, immunotherapy, recurrence

## Abstract

The survival time of patients with early clear cell renal cell carcinoma (ccRCC) is fairly long, but 20% to 30% of patients with localized tumors experience relapse, and the effect of IFN-α on survival has not been well studied in patients with early ccRCC. In this study, 208 patients with early ccRCC were treated with surgery, and 54 of the patients received IFN-α as adjuvant therapy. The remaining 115 patients were treated with surgery but not with IFN-α therapy. The primary endpoint was the recurrence rate, 20.37% (11/54) and 33.04% (38/115) in the IFN-α and surgery-only group, respectively. The secondary endpoint was progression-free survival (PFS), which was 123.70 (95% CI: 107.18–140.22) months for the IFN-α group, and 95.80 (95% CI: 82.18–109.42) months for the non-IFN-α group; this difference was significant (*P* < 0.05). The main side effects were pyrexia (61.11%), muscle pain (24.07%), malaise (9.26%), anorexia (5.56%), hepatic dysfunction (3.70%) and renal dysfunction (1.85%). Moreover, a multivariate regression identified older age, higher BMI index and smoking as significant and independent predictors of decreased PFS (*P* < 0.05). Overall, IFN-α therapy significantly improved PFS in Chinese patients with early ccRCC and was an independent prognostic factor (*P* < 0.05). In conclusion, our study showed that adjuvant IFN-α therapy decreased the recurrence rate and prolonged PFS in patients with ccRCC. Thus, this treatment may help clinicians to select a better treatment modality and better predict survival in these patients.

## INTRODUCTION

Renal cell carcinoma accounts for 2–3% of all new cancer cases [1], and ccRCC is the most common subtype accountings for 70–80% of all renal cancers. At present, the incidence of renal cell carcinoma among all cancers worldwide is growing 2.5% each year [2], and nephrectomy remains the preferred treatment for early ccRCC. However, 20% to 30% of patients with localized tumors experience relapse after surgical excision [3], and once metastasis occurs, the five-year survival rate is less than 10% [4]. However, effective adjuvant therapy for ccRCC after surgery has not yet been reported to successfully reduce the recurrence rate of ccRCC.

IFN-α is an immunotherapeutic agent generated by monocytes and macrophages, that has beneficial effects on human health in a variety of ways. For example, previous studies have shown that IFN-α activates the immune response [5], induces apoptosis [6], and directly inhibits the proliferation [7, 8] and differentiation of tumor cells [9]. It has also been proven effective as a multifunctional cytokine in pancreatic cancer cells [10]. Moreover, IFN-α has been used clinically and is recommended as a first-line systemic treatment for clear cell mRCC [11]. However, IFN-α has rarely been studied as a treatment for patients with ccRCC after surgery.

In this study, we collected clinical information from ccRCC patients. Patients were divided into two groups as a comparison. The final results were recurrence rate and the survival time of the two groups. Whether patients had IFN-α therapy was the only different factor between the two groups. A retrospective study was used to evaluate the effectiveness and safety of IFN-α in ccRCC. A follow-up survey was finished in June 2016. Survival curve and multivariate analysis were used for the prognosis of the two groups in ccRCC.

## RESULTS

### Baseline demographics and treatment tumor response

From May 1998 to June 2014, a total 208 ccRCC patients who underwent surgery at the General Hospital of Xinjiang Military Command were enrolled in this study. Thirty-nine patients were lost to follow-up in the study, and the remaining 169 patients included 122 males and 47 females. The mean maximum tumor diameter was 4.58 ± 2.13 cm (range 1.3–14.0 cm). Tumor size was classified as less than 7 cm (T1) or more than 7 cm (T2). All tumors were limited to the kidneys. The median age of the patients was 60.89 years. In order to compare the baseline characteristics between the experimental group and the control group, these patients were stratified by age (every 20 years). The median body mass index (BMI) was 24.55 ± 3.73 and the BMI was stratified as follows: less than 18.5 (underweight), 18.5 to 24.99 (normal), 25 to 27.99 (overweight), 28 to 31.99 (obese) and more than 32 (morbidly obese). The Eastern Cooperative Oncology Group (ECOG) performance status ranged from 0 (62.13%) to 1 (37.87%). The postoperative status and the comparison of baseline characteristics between the experimental group and the control group are shown in Table 1. These factors did not significantly differ between groups. After surgery, 54 patients had IFN-α therapy. The mean maximum tumor diameter of these patients was 5.17 ± 2.11 cm, their median age was 58.46 years, and their median BMI was 25.14 ± 3.48. The rates of recurrence in the experimental group are shown in Table 2. Specifically, an analysis of clinical information showed that the recurrence rate directly correlated with BMI and T stage (larger tumor) (*P* < 0.05). Therefore, the efficacy of IFN-α therapy may inversely correlate with BMI and T stage.

**Table 1:** Postoperative baseline demographic and clinical characteristics of Chinese patients with ccRCC

Characteristics	Number of patients in the experimental group(Percentage) *N* = 54	Number of patients in the control group (Percentage) *N* = 115	*P* value
Gender			
Male	37 (21.89%)	85 (50.30%)	0.466
Female	17 (10.06%)	30 (17.75%)	
Age (Years)			
0–20	0 (0.00%)	1 (0.59%)	0.251
21–40	3 (1.78%)	9 (5.33%)	
41–60	30 (17.75%)	45 (26.63%)	
61–80	19 (11.24%)	48 (28.40%)	
> 80	2 (1.18%)	12 (7.10%)	
BMI (kg/m^2^ )			
≤ 18.5	1 (0.59%)	12 (7.10%)	0.259
18.5–24.99	23 (13.61%)	52 (30.77%)	
25–27.99	20 (11.83%)	35 (20.71%)	
28–31.99	9 (5.32%)	12 (7.10%)	
≥ 32	1 (0.59%)	3 (1.78%)	
ECOG performance status			
0	34 (20.12%)	71 (42.01%)	0.878
1	20 (11.83%)	44 (26.04%)	
Smoking			
YES	18 (10.65%)	49 (28.99%)	0.250
NO	36 (21.30%)	66 (39.05%)	
Tumor size			
T1	45 (26.63%)	106 (62.72%)	0.082
T2	9 (5.32%)	9 (5.32%)	

**Table 2:** Recurrence and clinical features of IFN-α therapy for the Chinese patients with ccRCC

Characteristics	Number of recurrence-free patients (Percentage) *N* = 43	Number patients who developed recurrence (Percentage) *N* = 11	*P* value
Gender			
Male	27 (50.00%)	10 (18.52%)	0.143
Female	16 (29.63%)	1 (1.85%)	
Age (Years)			
0–20	0 (0.00%)	0 (0.00%)	0.602
21–40	3 (5.56%)	0 (0.00%)	
41–60	24 (44.44%)	6 (1.11%)	
61–80	15 (27.78%)	4 (7.41%)	
> 80	1 (1.85%)	1 (1.85%)	
BMI (kg/m^2^ )			
≤ 18.5	1(1.85%)	0 (0.00%)	0.029
18.5–24.99	14 (25.93%)	9 (16.67%)	
25–27.99	18 (33.33%)	2 (3.70%)	
28–31.99	9 (16.67%)	0 (0.00%)	
≥ 32	1 (1.85%)	0 (0.00%)	
ECOG performance status			
0	35(64.81%)	7 (12.96%)	0.237
1	8 (14.81%)	4 (7.41%)	
Tumor stage			
T1	40 (74.07%)	5 (9.26%)	0.001
T2	3 (5.56%)	6 (11.11%)	
Smoking			
YES	12 (22.22%)	6 (11.11%)	0.150
NO	31 (57.41%)	5 (9.26%)	

### Recurrence and death

By December 2016, 49 patients were confirmed to have developed recurrence by CT scans or other relevant examinations, and 16 patients had died because of recurrence-related complications. The range of follow up duration was 4.67–216.54 months, and the median duration was 110.82 months. The primary endpoint recurrence rate in the experimental group was 20.37%, which was significantly lower than that of the control group (33.04%) (*P* < 0.05). A univariate analysis was used to screen for potential prognostic factors, such as gender, age, BMI, ECOG performance status, tumor size and smoking status. The final follow-up results of the two groups are shown in Table 3. Specifically, recurrence directly correlated with a higher BMI, higher ECOG performance status, higher T stage and smoking (*P* < 0.05). Thus, these factors increase the recurrence risk and decrease the PFS of patients with ccRCC.

**Table 3:** Analysis of prognostic factors in Chinese patients with recurrent clear cell carcinoma

Characteristics	Number of recurrence-free patients (Percentage) *N* = 120	Number of patients who developed recurrence (Percentage) *N* = 49	*P* value
Gender			
Male	82 (48.52%)	40 (23.67%)	0.080
Female	38 (22.48%)	9 (5.33%)	
Age (Years)			
0–20	1 (0.59%)	0 (0.00%)	0.562
21–40	10 (5.92%)	2 (1.18%)	
41–60	55 (32.54%)	20 (11.83%)	
61–80	46 (27.21%)	21 (12.42%)	
> 80	8 (4.73%)	6 (3.55%)	
BMI (kg/m^2^ )			
≤ 18.5	3 (1.78%)	10 (5.92%)	0.006
18.5–24.99	56 (33.14%)	20 (11.83%)	
25–27.99	41 (24.26%)	14 (8.28%)	
28–31.99	17 (10.05%)	4 (2.37%)	
≥ 32	3 (1.78%)	1 (0.59%)	
ECOG performance status			
0	85 (50.30%)	23 (13.61%)	0.003
1	35 (20.71%)	26 (15.38%)	
Tumor stage			
T1	112 (66.27%)	40 (23.67%)	0.044
T2	8 (4.73%)	9 (5.39%)	
Smoking			
YES	40 (23.67%)	27 (15.98%)	0.009
NO	80 (47.34%)	22 (13.02%)	

### Prognostic factors for survival

**Figure 1:**
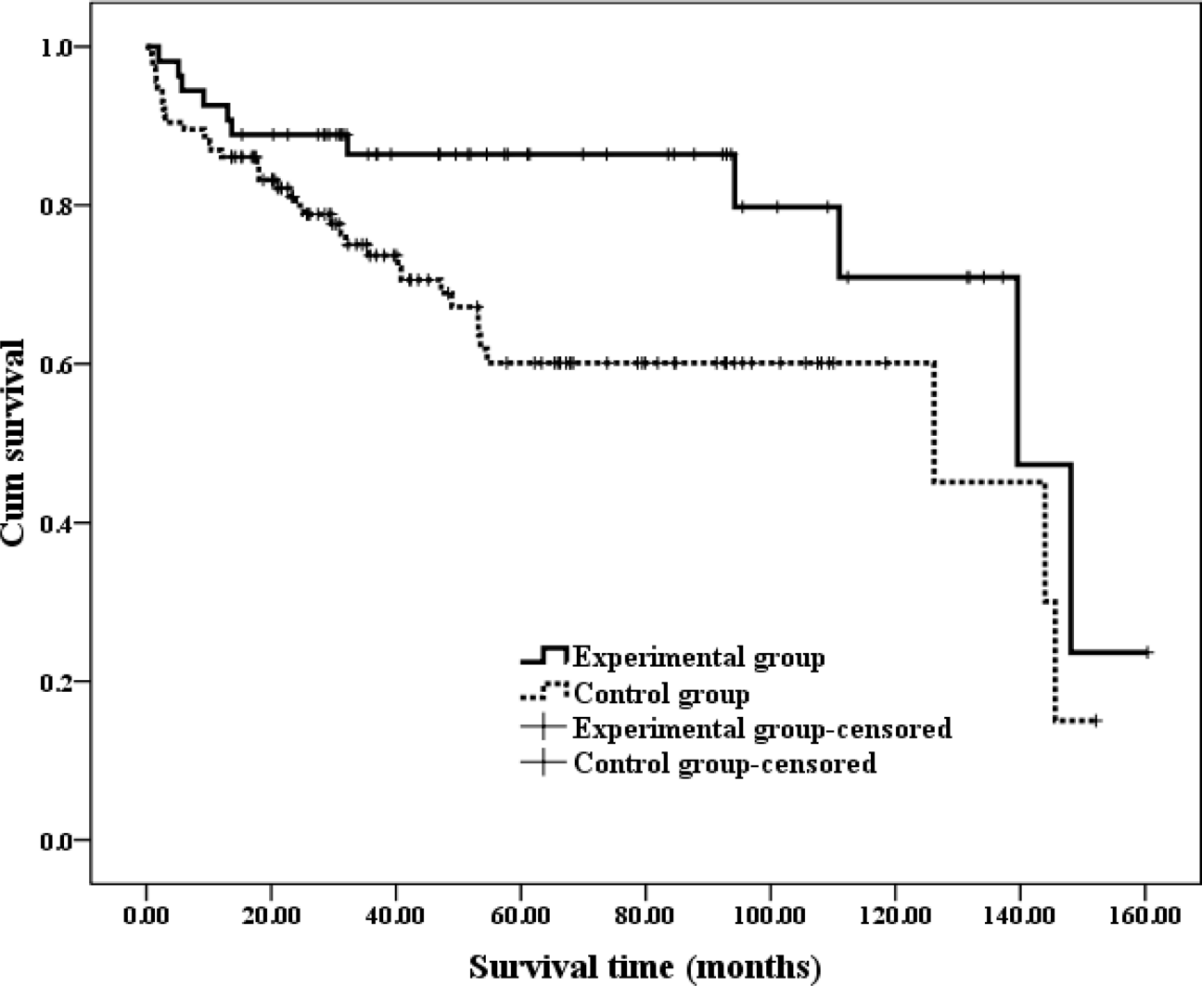
Kaplan-Meir estimates of PFS in different groups with ccRCC The median time of PFS was 139.57 (95% CI: 107.87–171.26) months in the experimental group and 126.19 (95% CI: 82.18–189.99) months in the control group.

**Figure 2:**
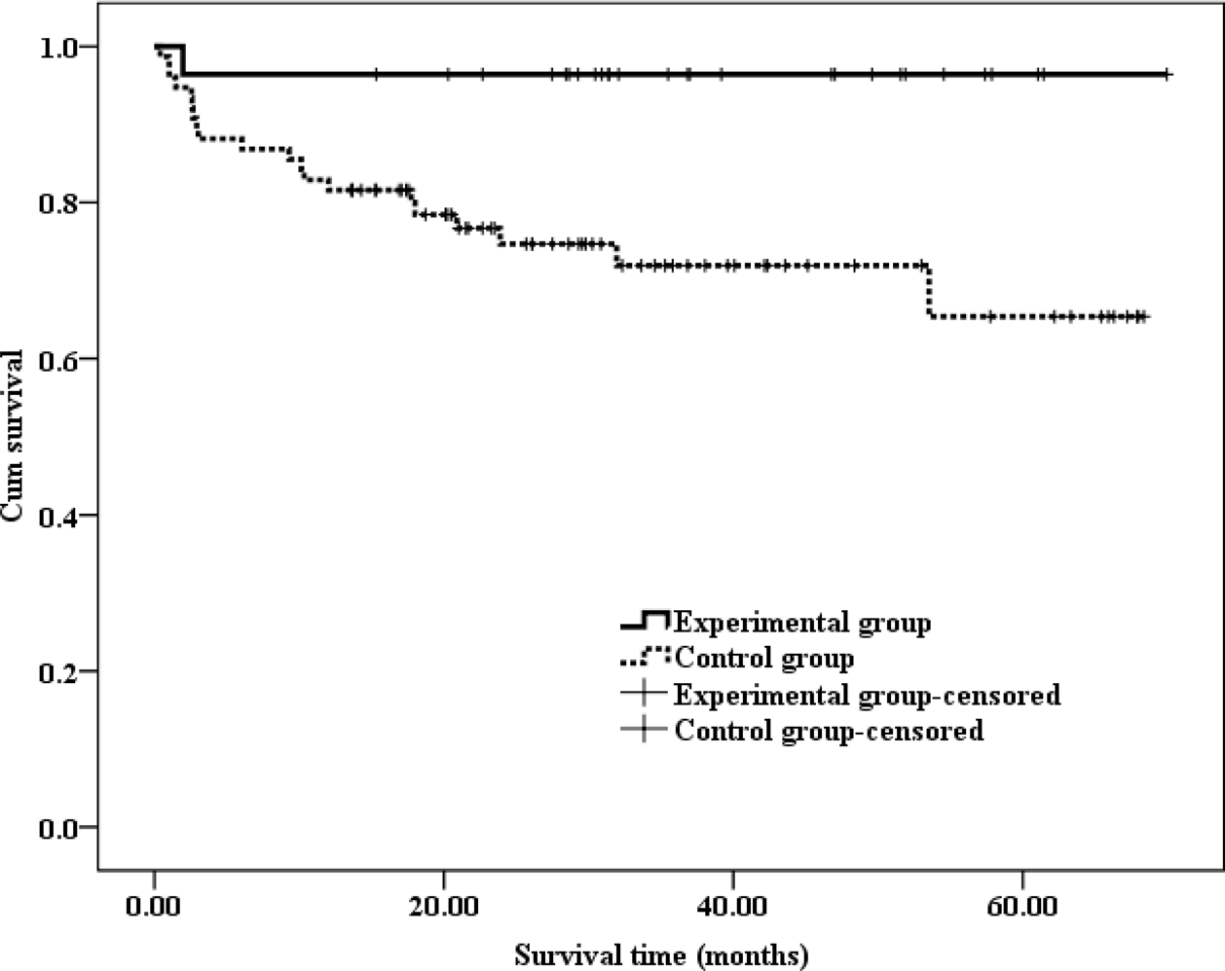
Kaplan-Meir estimates of 5-years PFS in patients with ccRCC The average PFS was 67.52 (95% CI: 62.85–72.19) months in the experimental group and 51.35 (95% CI: 44.99–57.71) months in the control group.

**Figure 3:**
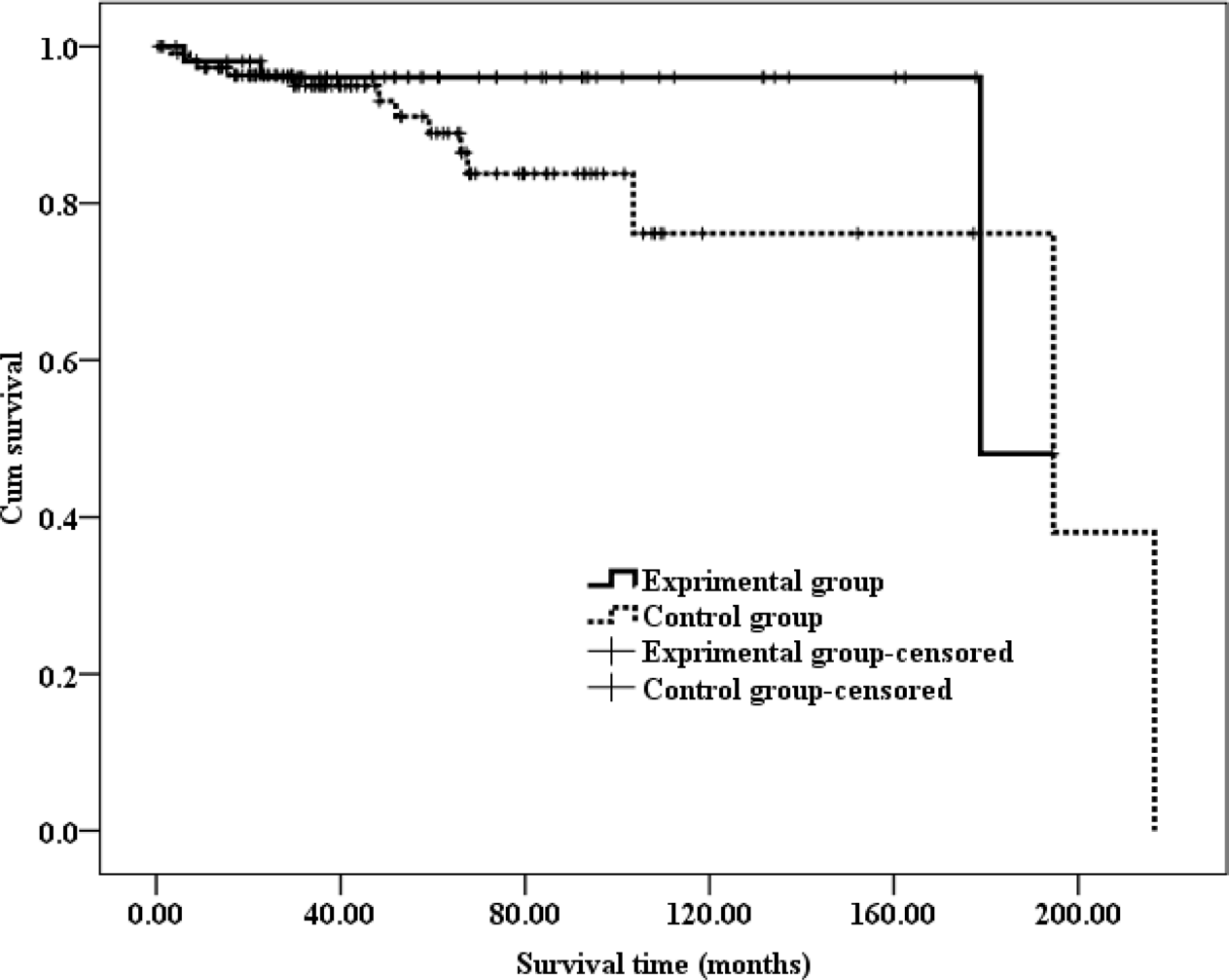
Kaplan-Meir estimates of OS in different groups with ccRCC The median OS was 178.79 (95% CI: 166.73–192.85) months in the experimental group and 171.84 (95% CI: 149.36–201.37) months in the control group.

Progression-free survival (PFS) was defined as the period from the date of surgery until the date of the last recurrence-free follow-up. The PFS of patients who had developed recurrence was defined as the period from the date of surgery until the date that the first disease recurrence was detected by medical imaging. The time from the initial surgery until death was defined as the OS. The results of a Kaplan-Meier analysis are presented in Figures 1–3.

Figure 1 shows that median PFS was 139.57 (95% CI: 107.87–171.26) months in the experimental group and 126.19 (95% CI: 82.18–189.99) months in the control group, and this difference was significant according to a log-rank test (*P* = 0.019).

The time at which patients had surgery differed between the groups. Specifically, therapy was more effective for patients who had surgery within 5 years, as indicated by the Kaplan-Meier analysis in Figure 2.

Figure 2 shows that the average PFS was 67.52 (95% CI: 62.85–72.19) months in the experimental group and 51.35 (95% CI: 44.99–57.71) months in the control group, and this difference was significant according to a log-rank test (*P* = 0.011)

However, the Kaplan-Meier analysis showed that the OS did not significantly differ between the two groups, as shown in Figure 3. Specifically, the median OS was 178.79 (95% CI: 166.73–192.85) months in the experimental group and 171.84 (95% CI: 149.36–201.37) months in the control group, and this difference was not significant according to a log-rank test (*P* = 0.196).

The Cox proportional regression model is shown in Table 4. A multivariate analysis revealed that IFN-α therapy was a significant and an independent predictor of increased PFS, whereas smoking, older age and a higher BMI were identified as significant predictors of decreased PFS (*P* < 0.05). The factors shown in Table 4 may also decrease the efficacy of IFN-α therapy. However, additional factors may affect the PFS of these patients, and these factors remain to be identified in future studies.

**Table 4:** Cox proportional regression model for ccRCC

Variable	Hazard Ratio (95% CI)	*P* value
IFN-α therapy	0.567 (0.378,0.851)	0.006
Smoking	1.868 (1.183,2.950)	0.007
Age group	0.523 (0.313,0.875)	0.014
BMI index	1.743 (1.187,2.560)	0.005
Diameter group	1.284 (0.859.1.920)	0.223
ECOG score	0.790 (0.457,1.367)	0.400
Gender	0. 852 (0.549,1.321)	0.474

### Adverse events

The statistical analysis of adverse events is shown in Table 5. Among the 54 patients who received IFN-α therapy, the main side effects included pyrexia (61.11%), muscle pain (24.07%), malaise (9.26%), anorexia (5.56%), hepatic dysfunction (3.70%) and renal dysfunction (1.85%). Symptomatic treatment was used when patients were not able to endure IFN-α therapy. All 54 patients finished at least one cycle of treatment, and none of the patients discontinued treatment due to adverse events.

**Table 5:** Adverse events in the experimental group

Variable	Any grade	≥ Grade 3
Pyrexia	33 (61.11%)	3 (9.09%)
Muscle pain	13 (24.07%)	1 (7.69%)
Malaise	5 (9.26%)	0 (0.00%)
Anorexia	3 (5.56%)	0 (0.00%)
Hepatic dysfunction	2 (3.70%)	0 (0.00%)
Renal dysfunction	1 (1.85%)	0 (0.00%)
Total cases	54 (100%)	4 (7. 41%)

## DISCUSSION

Surgical resection remains an effective treatment for clinically localized ccRCC, and partial nephrectomy is proven and well-established to produce oncologic outcomes comparable to those of radical nephrectomy [12]. Moreover, a recent study showed that treatment with partial rather than radical nephrectomy was associated with improved survival among Medicare beneficiaries with early-stage kidney cancer [13]. However, 20% to 30% of patients with localized tumors experience relapse after surgical excision. Results from a retrospective analysis indicated that in a subset of patients, relapses occur more than 5 years after surgery for their primary RCC [14]. Our study examined a period exceeding 10 years, which might provide more powerful evidence.

In our study, we found that the recurrence rate was lower in the experimental group than in the control group. Kaplan-Meier analysis showed that the PFS differed between the two groups, especially for patients who had surgery within 5 years. However, interferon-α therapy after nephrectomy for early ccRCC patients had not been investigated for many years. Recent research showed that IFN-α therapy had synergistic immunotherapeutic effects and inhibited the proliferation of mouse RCC Renca cells *in vitro* and *in vivo* [15]. Specifically, the dose-effect curve demonstrated that the addition of 4,000 IU/ml IFN-α significantly inhibited the viability of the Renca cells 48 hours after administration. Recently, a study discovered that structurally modified curcumin could promote the apoptosis of human renal cell carcinoma by inhibiting STAT phosphorylation [16]. Thus, the STAT pathway might become an emerging oncogenic target in the setting of ccRCC, melanoma, and other forms of cancer [16]. Indeed, recent studies showed that human ccRCC cell lines were sensitive to Jak2-STAT pathway activity induced by dimethoxycurcumin [17], and recent clinical data suggested that the effects of drugs regularly used to treat RCC might elicit their anti-tumor effects via targeting of the STAT pathway. These findings suggest that the STAT pathway might be induced by biological factors in ccRCC. Moreover, IFN signaling was shown to regulate the STAT pathway in 2014 [18], which further supported evidence that the IFN pathway contributes to complex diseases. Theoretically, the relationship between the IFN pathway and STAT expression may prevent the recurrence of ccRCC.

Patients treated with IFN-α as adjuvant therapy after surgery was first reported in 2004 [19]. In that study, 88 patients with RCC had IFN-α therapy (5 million units once, 5 times a week, 4 weeks per cycle). The survival rates based on the pre-administration pT stage showed that the progression inversely correlated with T stage (*P* = 0.0966). Duration of IFN-α administration tended to positively correlate with long-term survival (*P* = 0.3765). In this study, conclusions were obtained based on before-after trials (BAT), but follow-up periods were relatively short, which may result in drastic changes in disease progression. The mean duration of follow-up in this study was 70.46 months, which limits the comparability of the two studies. Our study adopted a two-arm design, which was suitable for our disease and ensured accurate results. However, a disadvantage in this study was bias, including information bias and confounding bias. To decrease information bias, our research adopted several methods to obtain information from patients, such as telephone calls, e-mails, letters, and even visits to their families. We also adopted a multivariate analysis to decrease confounding bias. Some risk factors of ccRCC were computed by multivariate analysis to exclude interference.

In China, the dose of IFN-α in guidelines suggest gradient increments. Therefore, patients received 3 million units of IFN-α in the first week. 6 million units in the second week, 9 million units in the third week. In our study, most patients finished IFN-α therapy on time. However, 4 patients paused treatment because of grade 3 or worse side effect, but these patients finished treatment after recovering, and none of the 54 patients dropped out of the study. Table 2 shows recurrence after IFN-α therapy directly correlated with BMI and T stage (larger tumor). Thus, 3 million units may not be suitable for patients with a higher BMI and higher T stage (larger tumor), but this hypothesis requires further study.

A study showed that smoking and being overweight were risk factors of RCC. The incidence of RCC increased with exposure to tobacco. The risk of RCC also increased with increasing number of smoking years. The incidence of RCC had a gender difference that might be related to smoking [20]. In our study, smoking, older age and a higher BMI predicted decreased PFS. Thus, smoking and a higher BMI may be both risk and prognostic factors in ccRCC, whereas older age may only play a prognostic role.

In conclusion, IFN-α-based immune therapy shows an effective extending of PFS in ccRCC patients, and may provide a new method to prevent the recurrence of ccRCC.

## MATERIALS AND METHODS

### Inclusion criteria

This retrospective study was approved by the review board of the General Hospital of Xinjiang Military Command. In total, 208 patients with early ccRCC who provided informed consent were screened between July 1998 and June 2014. The patients had at least one measurable lesion (over 10 mm in largest diameter), and baseline computed tomography (CT) scans were available for each patient. The CT scans were reviewed independently by at least two senior radiologists. Other relevant examinations included MRI, SPECT and PET/CT to confirm the tumor. All patients were pathologically diagnosed to ensure that the tumor was clear cell renal cell carcinoma. To be eligible for the study, patients were at least 18 years of age. The patients were free of other comorbidities and additional tumors. Their hepatic and renal functions were normal, and all tumors were limited to the kidneys. After screening, 39 patients were censored from this study, and the last follow-up for the remaining 169 patients was in December 2016. Specifically, 54 patients with early ccRCC treated with surgery who received IFN-α constituted the experimental group, and the remaining 115 patients, who were treated with surgery but not with IFN-α therapy, were enrolled in the control group.

### Treatment and follow-up

Patients had IFN-α therapy after surgery as treatment. 54 patients provided informed consent and received one dose of 3 million units IFN-α, three times per week, in four-weeks cycles. Treatment was continued for at least four cycles but stopped once unacceptable toxicity occurred. Once unexpected side effects, such as high fever, hepatic dysfunction or renal dysfunction exceeding grade 3 occurred, the dose of IFN-α was reduced or treatment was paused until the patients recovered.

Patients in the experimental group were followed up monthly during IFN-α treatment using complete blood count, serum electrolytes, liver function and renal function tests. After completing or stopping treatment, these patients were followed up every 3 months, the same as the control group. Assessments included CT scans or other relevant medical imaging examinations, physical examinations and routine laboratory tests.

### Outcomes and assessments

The primary end point was the recurrence rate, and the secondary end point was PFS (the period from the date of surgery until the date of the first medical imaging examination that indicated disease progression). Multivariate analysis of recurrence included prognostic factors, such as age, gender, tumor size, IFN-α therapy, smoking, ECOG performance status and BMI. The safety outcomes were the incidence of adverse events associated with the first administration of IFN-α (especially grade 3 or worse adverse events).

### Statistical analysis

The sample size in our study was computed by professional statisticians using the following formula: $n\;=\;2\bar{\text{p}}\bar{\text{q}}\;{\left({\text{Z}}_{\alpha }\;+\;{\text{Z}}_{\beta }\right)}^{2}/{\left({\text{p}}_{1}-{\text{p}}_{0}\right)}^{2}$, which yielded a sample size of 153.93. Our cohort was larger than this computed number, which ensures the credibility of our results. All statistical analyses were performed using SPSS software, version 17. Continuous variables, such as PFS and OS, are reported as medians and interquartile ranges. Categorical data, such as age, gender, tumor size, smoking and BMI, are presented as proportions. The follow-up duration was calculated using the Kaplan-Meier method, and the OS and PFS were estimated using the Kaplan-Meier method with a Rothman 95% CI and compared across groups using the log-rank test. The Cox proportional hazards model was used to evaluate the prognostic value of the investigated parameters. All *P* values were two-sided and were considered significant at < 0.05.
